# Auricular acupressure for insomnia in women with breast cancer: A systematic review and meta-analysis of randomized controlled trials

**DOI:** 10.1097/MD.0000000000041498

**Published:** 2025-02-14

**Authors:** Xin-Rui Huang, Min Xu, Yan Xu, Shu-Jie Wang, Fei-Lin Ni

**Affiliations:** a Department of Neurology, The First Affiliated Hospital of Zhejiang Chinese Medical University (Zhejiang Provincial Hospital of Chinese Medicine), Hangzhou, Zhejiang Province, China; b Office of the Dean, The First Affiliated Hospital of Zhejiang Chinese Medical University (Zhejiang Provincial Hospital of Chinese Medicine), Hangzhou, Zhejiang Province, China; c College of Nursing, Zhejiang University of Chinese Medicine, Hangzhou, Zhejiang Province, China; d Department of Breast Diseases, The First Affiliated Hospital of Zhejiang Chinese Medical University (Zhejiang Provincial Hospital of Chinese Medicine), Hangzhou, Zhejiang Province, China; e Department of Neurology, The First Affiliated Hospital of Zhejiang Chinese Medical University (Zhejiang Provincial Hospital of Chinese Medicine), Hangzhou, Zhejiang Province, China.

**Keywords:** auricular acupressure, auricular point, lymphoma, non-pharmacological therapy, sleep disorder, systematic review, Traditional Chinese Medicine therapy

## Abstract

**Background:**

Breast cancer as the malignant tumor with the highest incidence and mortality rate among the global female population. Insomnia is a common complaint in breast cancer patients, more than one-third (38–47%) of breast cancer patients suffer it. Auricular acupressure (AA), a non-pharmacological therapy, has been used in the studies to intervene in insomnia in breast cancer patients. The objective of this systematic review and meta-analysis is to investigate the efficacy and safety of AA therapy in intervening with insomnia in breast cancer.

**Methods:**

A systematic literature search was performed for 10 databases up to January of 2024 to identify randomized control trials (RCTs). The methodological quality of RCTs was assessed independently using the Cochrane Handbook for Systematic Reviews of Interventions. The quality of evidence was evaluated with the Grading of Recommendations Assessment, Development and Evaluation approach. Data were screened and extracted independently using predesigned forms. The meta-analysis was conducted using RevMan 5.3 software, *P*-value < .05 means statistically significant.

**Results:**

This review included 15 studies from 3 different countries with a total of 1125 adult participants. The pooled results showed that AA significantly in improving sleep quality (mean difference [MD] = ‐3.36, 95% confidence interval [CI]: [‐4.65, −2.07], *P* < .001) and life quality (MD = ‐7.82, 95% CI: [‐14.76, ‐0.88], *P* = .03). Based on data from sleep monitoring devices, AA was valuable for improving sleep efficiency (MD = ‐3.63, 95% CI: [‐4.19,-3.07], *P* = .03) in breast cancer patients. Adverse events were reported in 5 RCTs. Common adverse reactions include auricular skin allergic reaction (10/259, 3.9%), bruising (7/259, 2.7%), pain (3/259, 1.2%), and local pressure ulcers on the auricular points (2/259, 0.8%). The evidence grade was moderate because of the substantial heterogeneity among studies. Heart, Shenmen, and Subcortex were the 3 most numerous auricular points, with a total share of up to 71.70%.

**Conclusion:**

This systematic review and meta-analysis demonstrates the efficacy and safety of AA in intervening insomnia in breast cancer patients, providing a basis for the selection of clinical auricular points. However, the high-quality RCTs in existence are not enough, and more rigorous trials are needed to identify the efficacy of AA and insomnia.

## 1. Introduction

Worldwide, breast cancer is the most common fatal cancer in women and the second leading cause of cancer death after lung cancer,^[1]^ and approximately 685,000 women die of breast cancer in 2020, accounting for approximately 15.5% of all cancer deaths in the world.^[2]^ As the largest and most populous upper-middle-income country, 416,371 Chinese women were diagnosed with breast cancer in 2020, accounting for 18% of global breast cancer diagnoses. Meanwhile, China has the largest number of breast cancer deaths, accounting for approximately 17.1% of all cancer deaths, followed by the USA, which accounted for 6.2% of breast cancer deaths in the world.^[2,3]^ However, the actual number may be underestimated as China’s cancer registries only cover 31.5% of the country’s entire population.^[3,4]^

With rapid advances in the field of medical oncology, a review of breast cancer mortality trends reveals that the age-adjusted breast cancer mortality rate in the United States declined by more than 40% from 1988 to 2018.^[5]^ Significant progress has been made in the treatment of breast cancer.^[6]^ However, due to surgery, radiotherapy, chemotherapy and other treatment measures produce many adverse effects on the physiology and psychology of patients with breast cancer. How to improve the quality of life of breast cancer patients has become a key concern of medical personnel.^[7–9]^ Among them, insomnia is a very important clinical symptom in breast cancer patients.

Insomnia is an increasingly widespread phenomenon in the population, especially for women. This gender difference may partially explain the higher rate of insomnia in breast cancer patients. Cancer patients have 3 times the rate of insomnia compared to the general population, with breast cancer patients having the highest rate of insomnia.^[10–12]^ More than one-third (38–47%) of breast cancer patients have significant insomnia symptoms.^[13,14]^

Pharmacologic treatment is the most widely used treatment for insomnia, but the drug side effects have been confirmed, including perceived risk of dependence and tolerance, dizziness and excessive sedation.^[15–18]^ Patients have also expressed a preference for non-pharmacological treatments to improve their sleep quality.^[19]^ With advantages such as low side effects, efficacy and safety, external Chinese medicine treatment has gradually become popular around the world. Many scholars have done relevant research on the external therapeutic intervention of Traditional Chinese Medicine (TCM) for insomnia.^[20–24]^ Auricular acupressure (AA) is stimulated by placing plant seeds or magnetic beads on specific points on the ear and applying pressure to them, which is an effective method of external treatment in TCM with a history of >2500 years.^[25,26]^ Globally, several studies have explored the efficacy of AA on insomnia in breast cancer patients. However, there is a lack of evaluation of the efficacy of AA in intervening with insomnia symptoms in breast cancer patients. Therefore, the purpose of this systematic review and meta-analysis is to investigate the efficacy of AA therapy in intervening with insomnia in breast cancer.

## 2. Methods

### 2.1. Study design and search strategy

This systematic review and meta-analysis searched the database for articles related to AA intervention for insomnia symptoms in breast cancer patients among Chinese and English language. Various databases including PubMed, EMBASE, Cochrane Central Register of Controlled Trials, CINAHL, Web of Science, Nursing Reference Center Plus, China National Knowledge Infrastructure, Wanfang Database, Chongqing VIP Information, Sinomed, and the search time frame is from the establishment of the repository to January 2024. This study follows the PRISMA guidelines. The protocol of this systematic review was registered on PROSPERO with the registration number CRD42024495430. We developed a search strategy based on the PICOS framework, the search strategy consisted of 3 components: clinical condition, intervention, and study type is randomized clinical trial. The search strategy is: (((auricular point acupressure OR ear acupressure OR AA OR auricular pressure OR auricular point sticking OR auricular plaster therapy OR auricular therapy)) AND ((breast cancer OR breast tumor OR breast neoplasms) AND (sleep initiation and maintenance disorders OR insomnia OR sleep disorders OR sleep disturbance))). To increase the likelihood of finding relevant empirical studies, a review of other sources (i.e., references included in the study and systematic reviews of published articles).

Two of us were independently responsible for searching the literature and screening it by reading the abstracts and full text of the articles. Articles published in the English or Chinese language were included if they were randomized control trials (RCTs) investigating the association of AA for insomnia with breast cancer. Eligible interventions were AA regardless of auricular acupuncture. The comparison could be between sham or placebo AA and medication or usual care without additional intervention. Sleep quality was the primary outcome indicator, and secondary outcome indicators were quality of life, depression, and anxiety.

### 2.2. Data extraction and quality assessment

Two authors independently extracted data using predesigned forms and assessed quality using the Cochrane Collaboration tool for assessing risk of bias. Publication information (first author, year, country), participant characteristics and study design (sample size, group assignment, control condition), AA protocol (acupoint selection, treatment session, duration, and frequency), time points for assessments, and the results of each outcome were extracted for each study. Each RCT was assigned a low, high, or unclear risk of bias for 6 specific domains (random sequence generation, allocation concealment, blinding of participants and outcome assessment, incomplete outcome data, selective reporting, and other potential bias). Disagreements were resolved through discussion.

The primary outcome variable was the total score from the most commonly used subjective self-report sleep questionnaires, Pittsburgh Sleep Quality Index (PSQI). The secondary outcomes of interest included Functional Assessment of Cancer Therapy-Breast, the severity of depression/anxiety, Hospital Anxiety and Depression Scale, sleep information recorded with Actiwatch, or sleep quality assessed with other validated questionnaires.

### 2.3. Statistical analysis

The meta-analysis was performed using Cochrane Collaboration RevMan 5.3. The continuous data were presented with the mean difference (MD) and 95% confidence interval (95% CI). The dichotomous data were presented using relative risk with 95% CI. We pooled the results of all studies to assess the differences of efficacy between interventions. The heterogeneity among included studies was assessed by using the *I*^2^ statistics, with a *P*-value < .05 indicating the presence of heterogeneity. Publication bias was estimated with a funnel plot.

## 3. Results

### 3.1. Characteristics of the included studies

**Figure 1.**
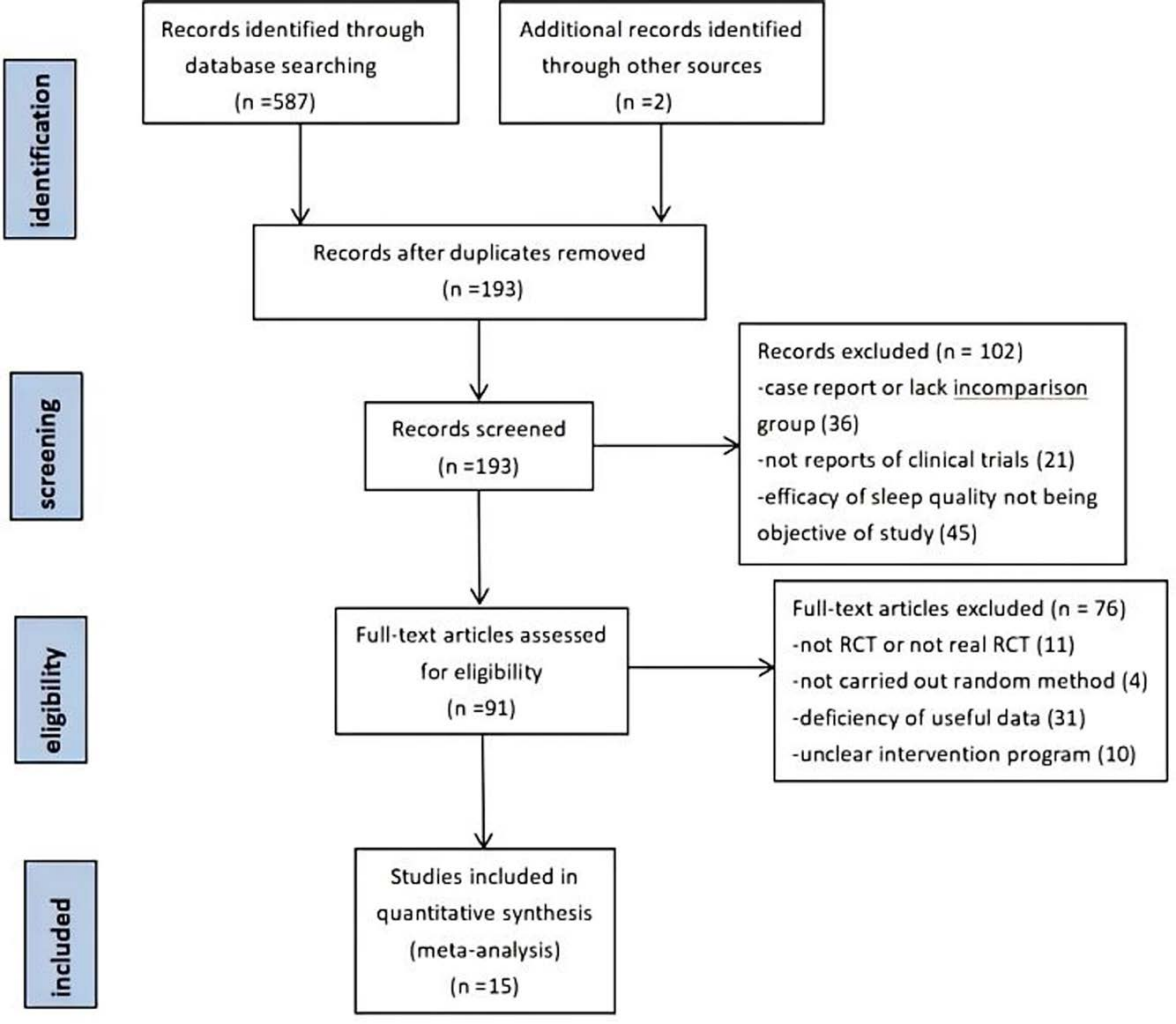
PRISMA flow diagram.

A total of 15 papers^[20,27–40]^ were included, and the specific screening flow diagram is shown in Figure 1. A total of 15 randomized controlled trials with 1125 patients were included. The summarized characteristics are listed in Table 1. Four studies were published in English language, and 9 studies were published in Chinese language. Thirteen RCTs originated from China, 1 RCT was from the Korea, and 1 RCT was from the United Kingdom. Sample size ranged from 5 to 152.

**Table 1:** Information of the included studies.

1st author	Year, country	Study design (intervention/control)	Sample size (intervention/control)	Intervention (regimen)	Control (regimen)	Intervention period	Sleep-related outcomes	Adverse events (intervention/control)	Acupuncture point selection
Shengmin Liu^[27]^	2023, China	AA/Usual care	99 (49/50)	AA (3 times/d, 5 min each time, 7 d/session, 4 sessions totally)	Usual care	4 weeks	PSQI, SDECMS	N.R.	Heart, Shenmen, Subcortex
Jialing Zhang^[20]^	2023, China (Hong Kong)	AA + active acupuncture/Sham acupuncture	138 (69/69)	AA + active acupuncture (12 sessions given twice weekly)	Sham acupuncture	6 weeks	ISI, PSQI, FACT-B, HADS, Actiwatch (SOL, WASO, TST, SE), BFI, BPI-SF, FACT-B	Bruising [13.0% (6/69)]/ auricular skin allergic reaction [5.8% (4/69)]	Heart, Shenmen, Sympathetic
Jianyi Huang^[28]^	2022, China	AA + herbal treatment/herbal treatment	72 (36/36)	AA + herbal treatment (4–6 times/d, 2–3 min each time, binaural alternation in 5-day cycles)	Herbal treatment	8 weeks	PSQI, SDECMS, HAMA, HAMD, hormone testing	Auricular skin allergic reaction [2.8% (1/36)]; pain in the auricle[5.6% (2/36)]	Heart, liver, spleen, Shenmen, endocrine, sympathetic, subcortex
Yuyan Wang^[29]^	2022, China	AA/health education	68 (34/34)	AA (4 times/d, 3 min each time, 7 d/session, 3 sessions totally, binaural alternation twice a week)	Health education	3 weeks	Actiwatch (TB, TST, SE, WASO), PSQI, FACT-B	Local pressure ulcers on their ear points [2.9% (2/68)]; Pain and minor nausea when receiving pressure on the sympathetic acupoint for the first time [1.5% (1/68)]	Heart, Shenmen, subcortex
Xiaoya Fa^[30]^	2022, China	AA + sleep patch acupressure/usual care	80 (40/40)	AA + Amiens paste (N.R.)	Usual care	4 weeks	PSQI, SF-36	N.R.	N.R.
Jialing Zhang^[31]^	2021, China (Hong Kong)	AA + EA/ wait-list	30 (15/15)	AA + EA (2 times per week, 6 weeks/session)	Wait-list	6 weeks	ISI, PSQI, HADS, FACT-B	Auricular skin allergic reaction [6.7% (1/15)]/auricular skin allergic reaction on auricular point [6.7% (1/15)]; Bruising [6.7% (1/15)]	Heart, Shenmen, sympathetic
Dongmei Zhao^[32]^	2020, China	AA + emotional care/usual care	152 (76/76)	AA + emotional care (pressure every 4 h, 1 min each time, 5 alternations/session, alternation 2–3 times a week)	Usual care	3 weeks	PSQI	N.R.	Heart, Shenmen, sympathetic, subcortex, endocrine
Xueying Li^[33]^	2019, China	AA + estazolam/estazolam	80 (40/40)	AA + estazolam (3 times/d, 5 min each time, binaural alternation everyday)	Estazolam	2 weeks	PSQI	N.R.	Heart, Shenmen, sympathetic, subcortex, endocrine, spleen, liver
Zhihao Zeng^[34]^	2019, China	AA + herbal treatment/ herbal treatment	42 (21/21)	AA + herbal treatment (3 times/d, 2 min each time, binaural alternation per 4 days, 14 d/session, 2 sessions totally)	Herbal treatment	4 weeks	PSQI	Auricular skin allergic reaction [14.3% (3/21)]	Heart, Shenmen, sympathetic, occiput, subcortex, spleen, liver
Hyeon^[35]^	2019, Korea	AA/SAA	41 (20/21)	AA (6 times per week, 1min each time, binaural alternation per 6 days)	SAA	6 weeks	PSQI (Korea Version), Fitbit tracker (TST, SE, SOL), Blood cytokines (cortisol associated)	N.R.	Heart, Shenmen, occiput, anterior lobe
Jia Lin^[36]^	2018, China	AA/usual care	60 (30/30)	AA (3–5 times per week, 3–5 min each time, binaural alternation per 3 days)	Usual care	10 days	PSQI	N.R.	Heart, Shenmen, sympathetic, subcortex, kidney
Jiahua Wu^[37]^	2017, China	AA + acupressure/usual care	80 (40/40)	AA (4 times per day, 3–5 min each time, binaural alternation per 3 days)	Usual care	6 days	PSQI	N.R.	Heart, Shenmen, sympathetic, subcortex, liver
Xiaoyan Qin^[38]^	2015, China	AA + herbal treatment/estazolam	88 (45/43)	AA (3 min each time, binaural alternation per 3–5 days, 10 d/session)	Estazolam	10 days	PSQI	N.R.	Heart, Shenmen, sympathetic, subcortex, spleen, kidney
Hughes^[39]^	2015, UK	AA/blank control	5 (3/2)	AA (1 min each time, binaural alternation per 7 days)	Blank	5 weeks	PSQI; MYCaW	N.R.	Heart, liver, kidney, subcortex
Yu Wang^[40]^	2014, China	AA/usual care	90 (45/45)	AA (3 times per day, 2 times per week, 8 times/session, binaural alternation)	Usual care	4 weeks	PSQI	N.R.	Heart, Shenmen, subcortex, endocrine, occiput

AA = auricular acupressure, BFI = Brief Fatigue Inventory, BPI-SF = Brief Pain Inventory-Short Form, EA = electroacupuncture, FACT-B = Functional Assessment of Cancer Therapy-Breast Cancer, HADS = Hospital Anxiety and Depression Scale, HAMA = Hamilton Anxiety Scale, HAMD = Hamilton Depression Scale, ISI = Insomnia Severity Index, MYCaW = the Measure Yourself Concerns and Wellbeing, N.R. = not reported, PSQI = Pittsburgh Sleep Quality Index, SAA = Sham Auricular Acupressure, SDECMS = Standard for Diagnosis and Efficacy of Chinese Medicine Syndrome, SE = sleep efficiency, SF-36 = Short Form 36 Health Survey Questionnaire, SOL = sleep onset latency, TB = time in bed, TST = total sleep time, UK = the United Kingdom, WASO = wake time after sleep onset.

### 3.2. Risk of bias

**Figure 2.**
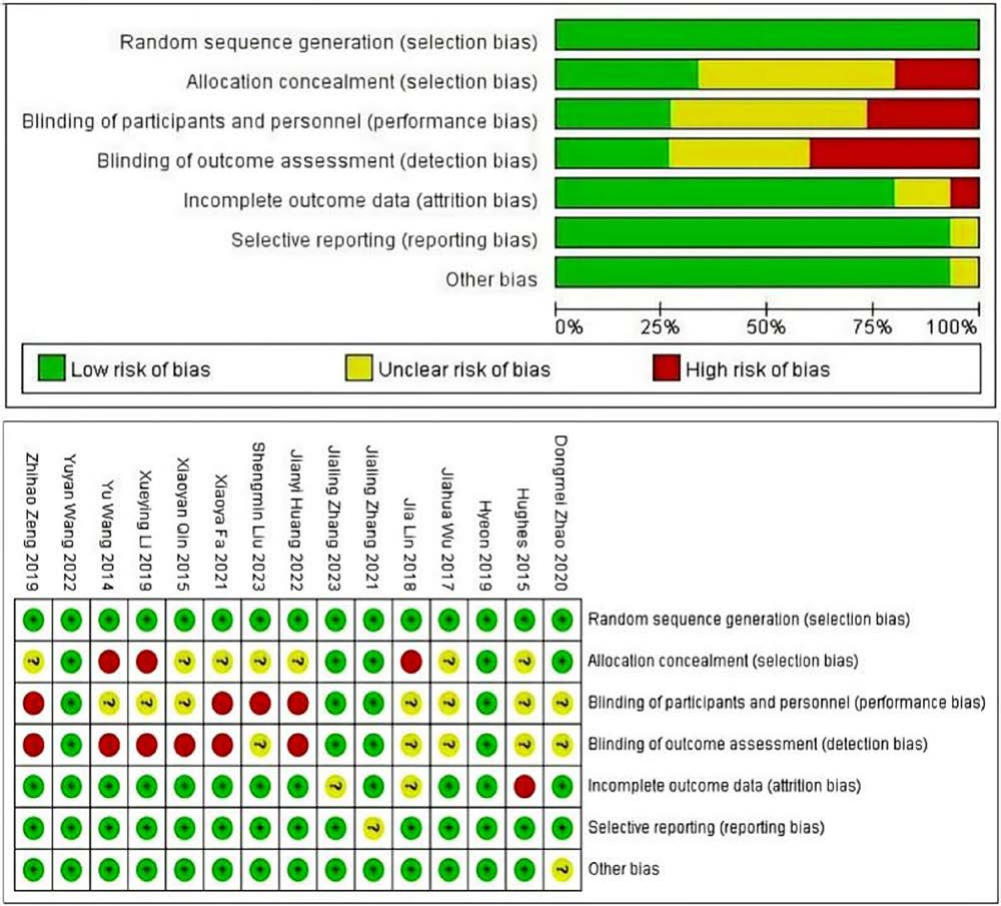
Evaluation of the risk of bias and applicability concerns.

**Figure 3.**
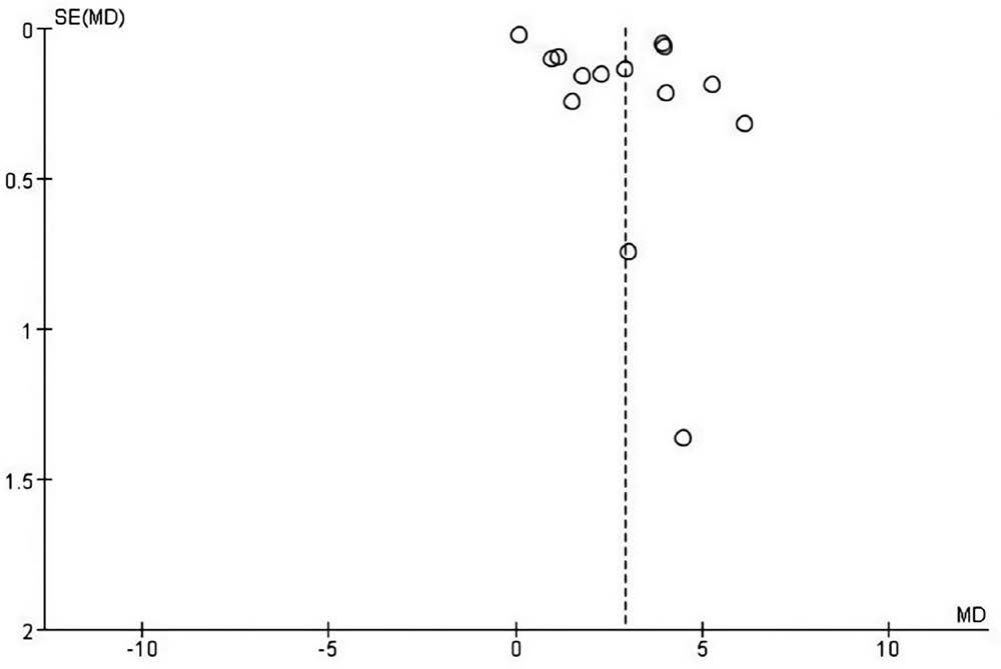
Funnel plot.

The risk of bias and applicability concerns evaluated according to Cochrane Collaboration tool is reported in Figure 2. All 15 studies reported randomization protocols with low risk of bias. 3 studies did not perform allocation concealment and 7 studies not clear. However, the highest risk of bias was the implementation of blinding, which was not implemented in 7 studies and was unclear in 8 studies. A funnel plot was drawn to determine publication bias, and the funnel plot showed that the distribution of literature roughly conformed to the symmetry of the 2 sides, see Figure 3.

### 3.3. The quality of evidence for each main outcome

This study included a total of 3 outcome measures, one of which included 3 sub outcome measures. The main outcome measure was PSQI, which was used in 15 articles. The other indicators consist of 3 articles. The project team members used GRADE’s scoring method to evaluate the risk of research bias, inconsistency, indirectness, and publication bias. An evaluation was conducted and ultimately provided a quality assessment result. The specific results are shown in Table 2.

**Table 2:** The quality of evidence for each main outcome.

Outcome indicators		Research quantity	Risk of research bias	Inconsistency	Indirectness	Inaccuracy	Publication bias	GRADE evidence quality
PSQI		15 RCTs	Low	None	None	Low	None	High
Fact-B		3 RCTs	Low	Medium	None	Medium	None	Medium
Outcomes of actiwatch	Wake time after sleep onset	3 RCTs	Low	Medium	None	Medium	None	Medium
	Total sleep time	3 RCTs	Low	Medium	None	Medium	None	Medium
	Sleep efficiency	3 RCTs	Low	Medium	None	Medium	None	Medium

### 3.4. Meta-analysis

#### 3.4.1. Outcomes related to insomnia

##### 3.4.1.1. Primary outcome indicator: PSQI

**Figure 4.**
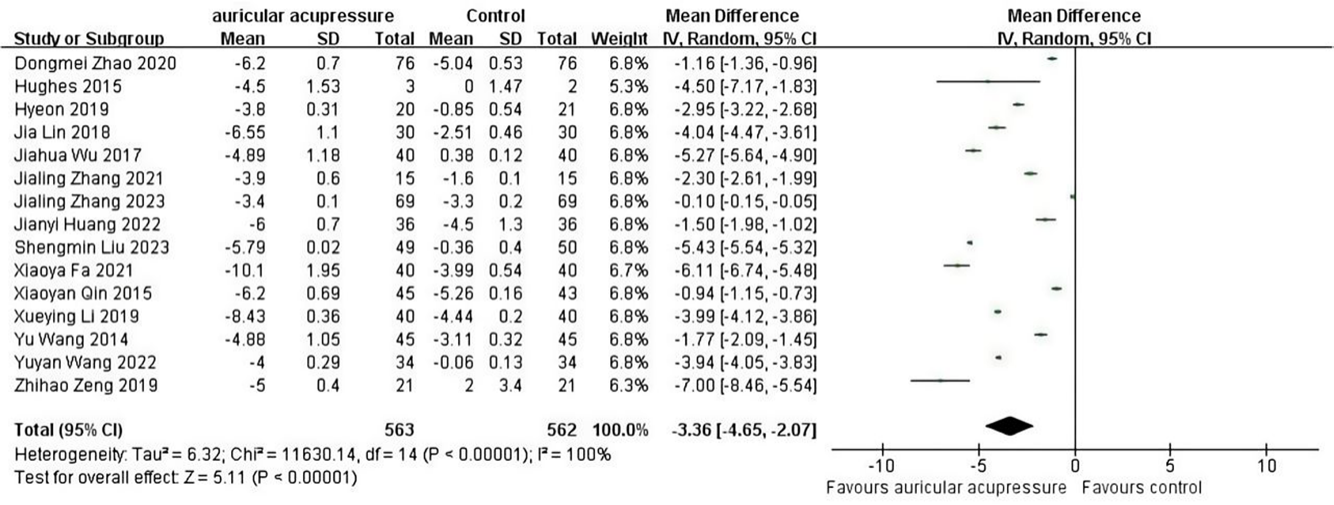
Forest map of PSQI. PSQI = Pittsburgh Sleep Quality Index.

**Figure 5.**
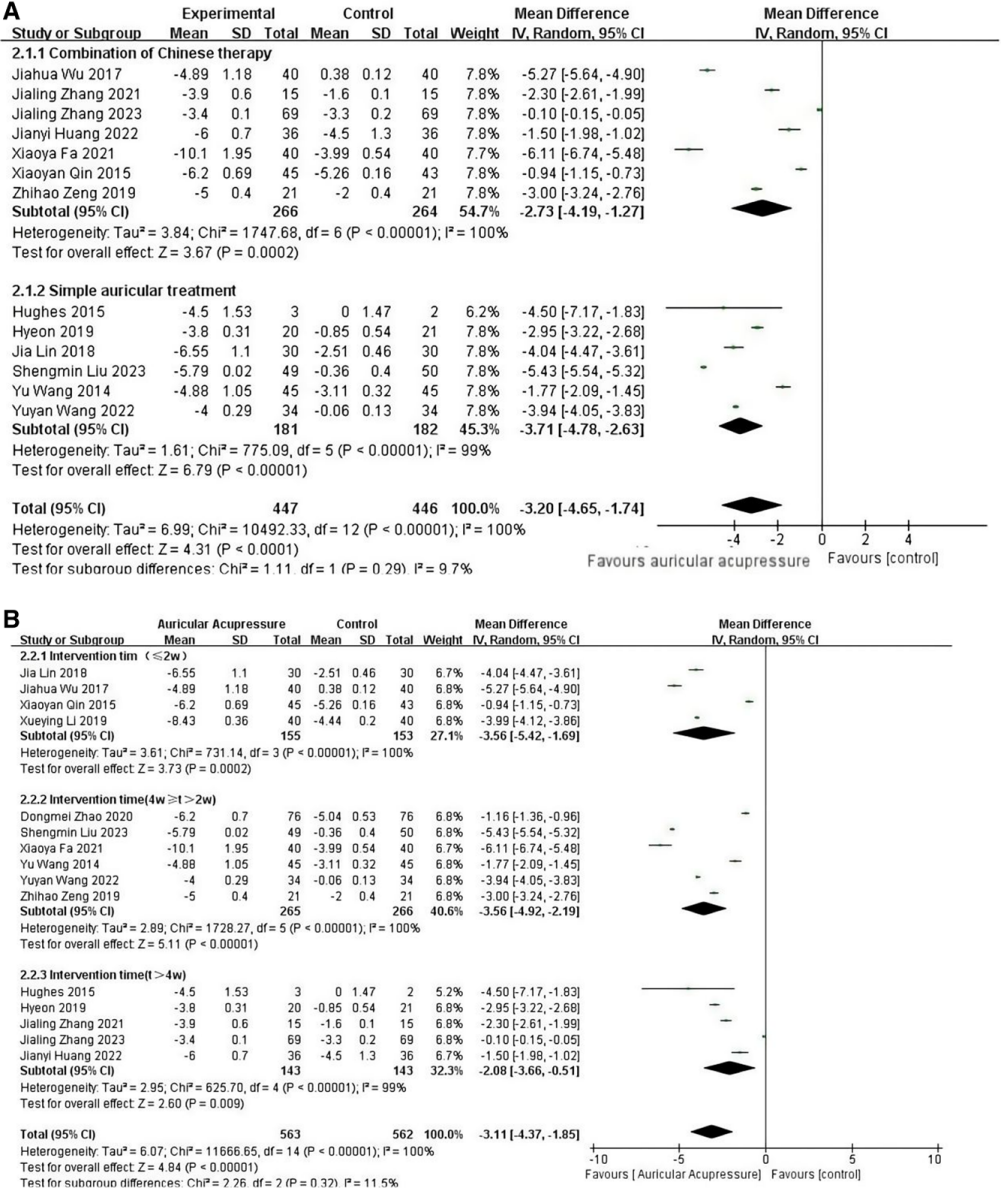
(A) Forest maps for PSQI subgroup analysis – modalities of intervention. (B) Forest maps for PSQI subgroup analysis – interventions times. PSQI = Pittsburgh Sleep Quality Index.

All 15 articles used the PSQI to assess sleep quality. The pooled results showed that AA significantly in reducing the index of PSQI, implying an improvement in sleep quality. (MD = ‐3.36, 95% CI: [‐4.65, −2.07], *P* < .001) (Fig. 4). Because of the high level of heterogeneity, sensitivity analyses were conducted to explore the sources of heterogeneity. However, there was no substantial change. According to the modalities of interventions, 15 RCTs were divided into 2 subgroups: AA alone (6 RCTs), and AA combined with other TCM therapies (7 RCTs). Subgroup analyses showed low heterogeneity (*I*^2^ = 9.7%, MD = ‐3.20, 95% CI: [‐4.65, −1.74], *P* < .001) (Fig. 5A). Meanwhile, 3 subgroups were divided based on the duration of the intervention using 2 and 4 weeks as boundaries, and subgroup analyses showed low heterogeneity (*I*^2^ = 11.5%, MD = ‐3.11, 95% CI: [‐4.37, −1.85], *P* < .001) (Fig. 5B). Whether the intervention was combined with other treatments in the observation group and the duration of the intervention may have contributed to the high heterogeneity.

### 3.5. Secondary outcome indicators

#### 3.5.1. Fact-B

**Figure 6.**
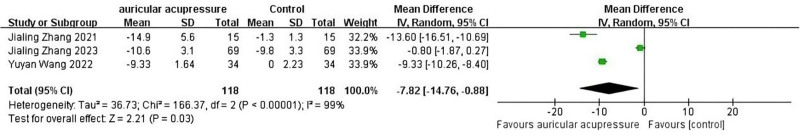
Forest map of Fact-B.

Three articles used the Functional Assessment of Cancer Therapy-Breast to assess improvements in breast cancer patients’ quality of life after the intervention. The results of the meta-analysis showed that AA was effective in improving the quality of life of breast cancer patients (MD = ‐7.82, 95% CI: [‐14.76, −0.88], *P* = .03). The result of the forest plot is shown in Figure 6.

#### 3.5.2. Outcomes of Actiwatch

**Figure 7.**
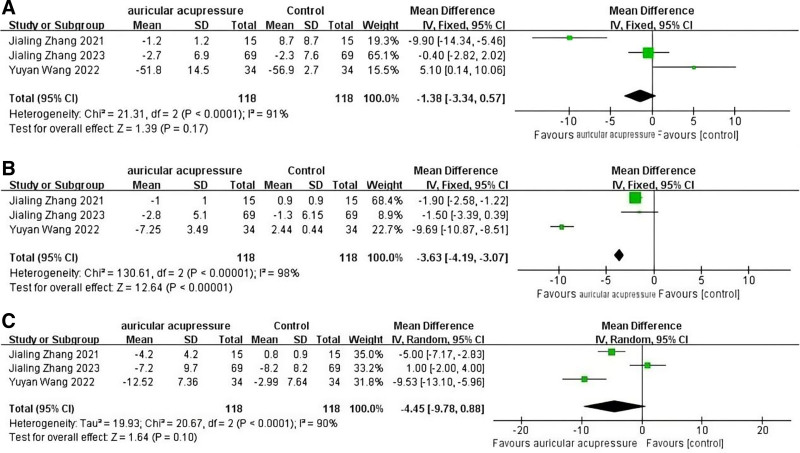
(A) Forest map of TST. (B) Forest map of SE. (C) Forest map of WASO. SE = sleep efficiency, TST = total sleep time, WASO = wake time after sleep onset.

Three articles used the wearable device Actiwatch for real-time monitoring of patients’ sleep status. Among them, wake time after sleep onset, total sleep time, and sleep efficiency were included in the meta-analysis. Another article,^[35]^ it also used a wearable device (Fitbit tracker) for the monitoring of sleep quality. It showed no statistical significance and did not present specific values in the original article, so this indicator was excluded from the meta analysis performed in this study. The results of meta-analysis showed that AA was valuable for improving sleep efficiency (MD = ‐3.63, 95% CI: [‐4.19, ‐3.07], *P* = .03) in breast cancer patients, but not for wake time after sleep onset (MD = ‐4.45, 95% CI: [‐9.78, 0.88], *P* = .10) and total sleep time (MD = ‐1.38, 95% CI: [‐3.34, 0.57], *P* = .17). The forest plots of the meta-analysis are shown in Figure 7.

### 3.6. Adverse events

Adverse event monitoring was only reported in 5 RCTs, but no mention of side effects in the other 10 RCTs. No serious adverse effects were noted in these studies. Zhang et al^[20]^ reported that adverse event occurred 6 cases in the AA group and 4 cases in the control group. Huang et al^[28]^ reported that adverse event occurred 3 cases in the AA group. Wang et al^[29]^ reported that adverse event occurred 3 cases in the AA group. Zhang et al^[31]^ reported that adverse event occurred 1 cases in the AA group and 2 cases in the wait-list group. In the study by Zeng et al^[34]^ no adverse event was happened in the control group, there were 3cases in the AA group. Common adverse reactions include auricular skin allergic reaction (10/259, 3.9%), bruising (7/259, 2.7%), pain (3/259, 1.2%), and local pressure ulcers on the auricular points (2/259, 0.8%). Rash and itching on the auricle and cheeks are clinical signs of the auricular skin allergic reaction.

### 3.7. Frequency analysis of selected auricular points

**Figure 8.**
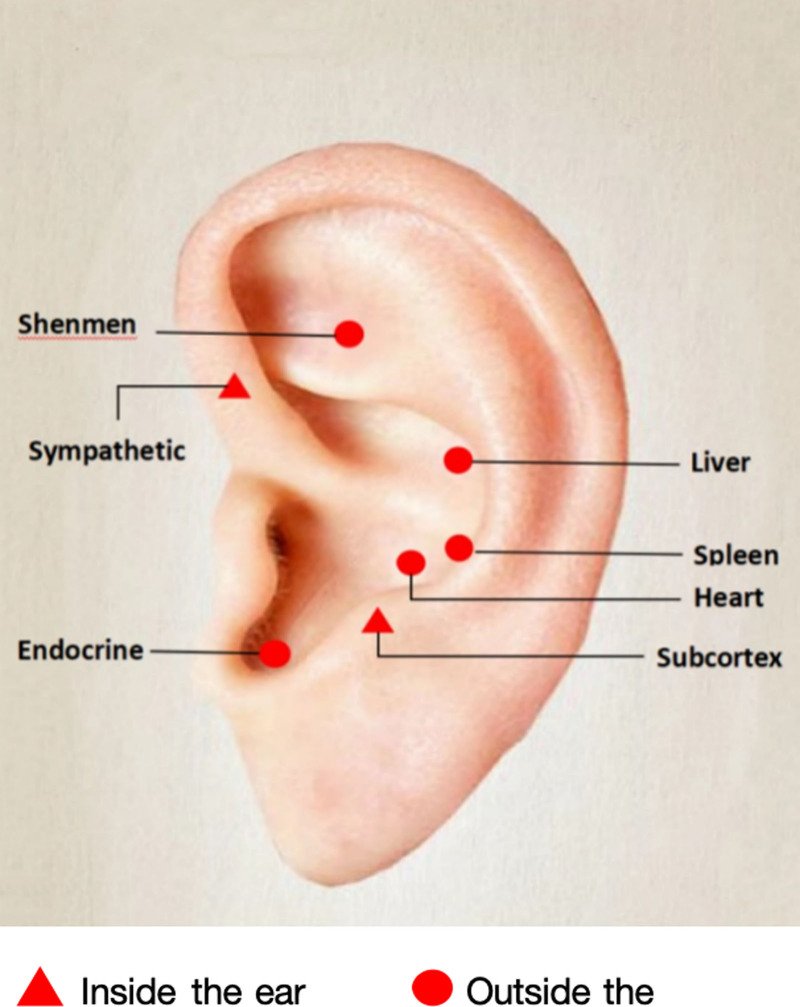
Schematic diagram of common auricular points.

Fourteen articles described the selection of auricular points, we counted a total of 10 auricular points involved, and the results showed that the Heart, Shenmen, and Subcortex were the 3 most numerous points, with a total share of up to 71.70%. The specifics of the auricular frequency analysis are detailed in Table 3. The localization of the auricular points on the corresponding ears is shown in Figure 8.

**Table 3:** The specifics of the auricular points frequency analysis.

Ear acupuncture point	Frequency (times)	Proportion (%)	Ear acupuncture point	Frequency (times)	Proportion (%)
Heart	14	100	Endocrine	4	28.57
Shenmen	13	92.86	Spleen	4	28.57
Subcortex	11	78.57	Occiput	3	21.43
Sympathetic	9	64.29	Kidney	3	21.43
Liver	5	35.71	Anterior lobe	1	7.14

## 4. Discussion

This systematic review and meta-analysis, we analyzed the associations between insomnia of breast cancer and AA. To the best of our knowledge, this is the first meta-analysis providing comprehensive insights into the effects between insomnia symptoms and AA therapy in breast cancer patients. We included 15 randomized controlled trials involving 1125 patients with breast cancer insomnia. The results of this study showed that AA therapy is effective in intervening insomnia symptoms of breast cancer patients. The adverse events caused by AA in the RCTs were not reported. This may be related to the small sample size. However, it is worth noting that AA is a noninvasive operation of external treatment of traditional Chinese medicine, with high safety.^[41]^ As is reported previously, AA can relieve neuronal excitability through facilitating the normalization of pathological hypersensitive reflex pathways connecting the ear microsystem and somatotopic brain, and regulating proinflammatory cytokines, such as IL-1b, IL-6, and TNF.^[42]^ This study confirms that AA is effective in treating insomnia in breast cancer patients. Although we did a subgroup analysis for the intervention modalities, we are not sure which is more effective when combined with other TCM treatment options compared to AA alone.^[43]^ This may be due to the high heterogeneity of the protocols using combined TCM intervention therapies in the RCTs. Of the studies we included, 7 RCTs combined other external TCM treatment, providing a rich combination of options. Three RCTs combined herbal treatments, 1 RCT combined acupressure, 1 RCT combined electroacupuncture, 1 RCT combined sleep patch acupressure, and 1 RCT combined acupuncture treatment. Obviously, we provide ideas to draw from the TCM combined treatment program. Our findings suggest that active AA therapy is more effective than Sham Auricular Acupressure, which is consistent with the research of many scholars.^[41,44,45]^

Multiple studies have shown that women have higher rates of insomnia than men, so hormone levels may respond to the efficacy of the intervention.^[46]^ J Huang et al^[28]^ explored the alteration of hormone levels in AA for the insomnia patients with breast cancer. However, his findings showed no statistical difference in the changes of any of the hormone types. This is inconsistent with the findings of other scholars.^[47,48]^ We hypothesize that the reason may be due to the fact that insomnia in breast cancer patients has a strong correlation with the disease itself, in addition to gender factors. In addition, the time point of the intervention of AA therapy may also have an effect. The observation time point of this clinical trial was at 8 weeks of intervention, which is relatively homogeneous, and further longitudinal studies with multiple time points, large samples, and multiple centers can be conducted in the future to explore the relationship between AA and hormone levels.

The choice of auricular points is a key point to perform AA. Data from the auricular points mentioned in the 15 RCTs yielded that 10 auricular points were selected for the treatment of insomnia. Among them, the Heart, Shenmen, Subcortex, and Sympathetic are some of the most important auricular points. Chinese medicine believes that the auricular points and the body’s internal organs correspond to the stimulation of specific auricular points can adjust the function of the corresponding organs.^[49]^ The main organs related to sleep are the heart, while the ear points corresponding to the heart are the Heart and Shenmen.^[50,51]^ The sympathetic point can regulate autonomic nerve, subcortex point can not only regulate cerebral subcortical autonomic nerve, but also regulate the endocrine system. By stimulating the specific auricular points, can calm the mind, replenish the heart and relieve neurasthenia, and then promote sleep.^[52]^

### 4.1. Study limitations and prospects

Since AA is a type of external therapy in Traditional Chinese Medicine, many of the randomized controlled trials we included were from China. In the future, we expect that more countries can try this therapy and conduct clinical trials to verify its efficacy. The overall quality of the literature was moderate due to the fact that the implementation of blinding may have been more difficult for the researcher. In the future, there is a need to improve the science and rigor of trial protocol design. Although some of the RCTs evaluate hormone levels and blood tests, overall there are fewer studies that include objective indicators of physiology.

## 5. Conclusion

The findings of this systematic review and meta-analysis suggest that, based on moderate-level evidence, AA may be associated with significant improvement in insomnia in breast cancer.

## Author contributions

**Conceptualization:** Xin-Rui Huang, Fei-Lin Ni, Min Xu.

**Data curation:** Min Xu.

**Formal analysis:** Xin-Rui Huang, Min Xu.

**Funding acquisition:** Fei-Lin Ni.

**Investigation:** Xin-Rui Huang.

**Methodology:** Xin-Rui Huang, Min Xu.

**Resources:** Xin-Rui Huang, Shu-Jie Wang.

**Software:** Shu-Jie Wang.

**Supervision:** Fei-Lin Ni, Xin-Rui Huang.

**Visualization:** Yan Xu.

**Writing – original draft:** Xin-Rui Huang, Yan Xu.

**Writing – review & editing:** Xin-Rui Huang, Yan Xu.
